# Maternal and Neonatal Factors Affecting Bone Mineral Content of Indonesian Term Newborns

**DOI:** 10.3389/fped.2021.680869

**Published:** 2021-05-25

**Authors:** Tunjung Wibowo, Neti Nurani, Janatin Hastuti, Alifah Anggraini, Rina Susilowati, Mohammad Hakimi, Madarina Julia, Mirjam Van Weissenbruch

**Affiliations:** ^1^Department of Child Health, Faculty of Medicine, Public Health, and Nursing, Universitas Gadjah Mada/Dr. Sardjito General Hospital, Yogyakarta, Indonesia; ^2^Department of Health Nutrition, Faculty of Medicine, Public Health and Nursing, Universitas Gadjah Mada, Yogyakarta, Indonesia; ^3^Department of Histology and Molecular Biology, Faculty of Medicine, Public Health and Nursing, Universitas Gadjah Mada, Yogyakarta, Indonesia; ^4^Department of Obstetrics and Gynaecology, Faculty of Medicine, Public Health and Nursing, Universitas Gadjah Mada, Yogyakarta, Indonesia; ^5^Department of Neonatology, Amsterdam University Medical Center Location VU University Medical Center, Emma Children's Hospital, VU University Amsterdam, Amsterdam, Netherlands

**Keywords:** neonatal bone mineral content, birth weight, gestational age, sex, maternal smoking

## Abstract

**Background:** Interactions between the genome and intrauterine environment can affect bone mineralization in newborns and even in adult life. Several studies show that intrauterine fetal bone mineralization or early postnatal bone condition influences the risk of osteoporosis in later life.

**Objectives:** To determine whole body bone mineral content (WB BMC) and factors that influence neonatal WB BMC in Indonesian term newborns.

**Subjects/Methods:** A cross-sectional study was conducted in Dr. Sardjito General Hospital, Yogyakarta, Indonesia. A total of 45 term, appropriate for gestational age (AGA) newborns were included in this study. BMC was assessed by dual-energy x-ray absorptiometry (DXA) in the first week of life. Weight (g), length (cm) and head circumference (cm) were measured at birth. Data on maternal characteristics were obtained from the maternal health records or reported by the mothers.

**Results:** WB BMC measured in the present study (mean ± SD: 33.2 ± 9.3 g) was lower than WB BMC of similar populations in developed countries. Multiple linear regression showed that birth weight, birth length, and gestational age had a positive association with WB BMC (*p* = 0.048, 0.017, and <0.001, respectively), while maternal cigarette exposure had a negative association with WB BMC (*p* = 0.012). Male infants had significantly higher of WB BMC than female (*p* = 0.025). These determinants contribute to 55% variability of WB BMC.

**Conclusions:** WB BMC in Indonesian term newborns is lower than populations in developed countries. Birth weight, length, gestational age, sex, and maternal cigarette exposure during pregnancy are significantly associated with WB BMC observed in Indonesian newborns.

## Introduction

Osteoporosis has a significant impact on a person's quality of life as well as being a massive economic burden for the country. In 1990, the worldwide annual direct and indirect burden of hip fracture was estimated at US $ 34.8 billion, rising to $ 131.5 billion by 2050 (at a rate of $ 21,000 per patient) (1). There is growing evidence of an interaction between the genome and the environment in the expression of several chronic diseases including osteoporosis (2). Data showed that intrauterine fetal bone mineralization or bone condition at birth will influence the risk of osteoporosis later in life (3).

Minton et al. (4) observed an association between gestational age and birth weight with the newborns' WB BMC in a Caucasian population in the United States (4). A study in Korea showed the influence of season of birth, cord serum 25-hydroxyvitamin D, maternal diabetes, alcohol consumption and maternal smoking during pregnancy on fetal bone mineralization (5).

A systematic review of the AGA-term infant population with predominantly Caucasian ethnicity found that the WB BMC value measured by Hologic DXA was 66.2 g (95% CI 65.4–67.05), while with Lunar DXA it was 78.9 g (95% CI 78.4–79.4) (2). It has been widely known that bone mass is influenced by race and ethnicity (6, 7). A study in a multiethnic population of prepubertal children using DXA showed ethnic differences in total body WB BMC (7). Research in seven Asian countries that aimed to assess bone health using heel ultrasound in adults aged 46–85 years indicated that Indonesian men and women had the poorest bone health (8). In addition, it was obvious that most pregnant women in Indonesia were Vitamin D deficient even though they live in a tropical country where sun exposure was abundant (9, 10).

With respect of cigarette smoking, Indonesia has one of the highest prevalence of active smokers in the world, with 55.8 % of men aged 10 years and over, compared to just 1.9 % of women of the same age (11). In view of these information, we aimed to assess the Indonesian term newborns' WB BMC and its associated factors.

## Methods

### Study Population

We enrolled 45 term infants hospitalized in the newborn nursery of Dr. Sardjito General Hospital from March 2018 to March 2019. Inclusion criteria were gestational age ≥ 37 weeks, appropriate for gestational age (AGA), and medically stable. Infants with major congenital abnormalities, bone disorders, gastrointestinal, or renal diseases were excluded.

### Maternal Characteristics

Pre-coded questionnaires were used to collect data on maternal characteristics consisting of maternal age (y), parity, education level, and cigarette exposure during pregnancy. Information on pre-pregnancy weight and maternal height were taken from the maternal health records or reported by the mothers. Pre-pregnancy maternal body mass index (BMI) was calculated as pre-pregnancy weight (kg) divided by height (m) squared. The Institute of Medicine (IOM) classification system was used to categorize BMI (kg/m^2^): underweight, <18.5; normal, 18.5 to <24.99; overweight, 25 to <29.99; and obese, ≥30 (12). Maternal hemoglobin level (mmol/l) before delivery was taken from medical records. Hemoglobin levels were measured using Sysmex XN-1000 (Sysmex, Kobe, Japan) with flow cytometry method (13). Anemia in pregnancy was defined as hemoglobin level <6.83 mmol/l (= 11 g/dl) (14).

### Newborn Characteristics

Newborn characteristics consisted of gestational age, gender, and the anthropometric measurements, that is, weight, length and head circumference, were obtained within 24 h after delivery. Gestational age was defined using the first trimester ultrasonogram or Dubowitz score, when the ultrasonogram information was not available. Weight in grams (g) was measured using calibrated electronic digital scale (Seca 727, Hamburg, Germany) to the nearest 0.1 g. Length was measured using a standard length-measuring board (Seca GmbH & Co. Hamburg, Germany) by two trained nutritionists to the nearest 0.1 centimeter (cm). Head circumference was measured with a non-stretch measuring tape to the nearest 0.1 cm. Infants were classified as AGA if their birth weight were ≥ 10th and <90th percentile for gestational age, using PediTools Fenton 2013 (15).

WB BMC, WB BMC except head, trunk BMC and legs BMC were measured using dual-energy X-ray absorptiometry (DXA; Prodigy: GE-Lunar Corp., Madison, WI, USA) as describe elsewhere (16). Assessment of BMC in newborns using DXA is an accurate method with less radiation exposure and fast scan time (2, 17). Calibration was performed using a calibration block supplied by the manufacturer consisting of tissue-equivalent material with three bone-simulating chambers. For quality control, the baby's body weight measured by the DXA were re-checked using a digital scale (SECA 727). The difference of body weight measurement by DXA and digital scale in this present study was 3.5%.

The BMC was assessed within the first week of life. The mother was asked to breastfeed the baby before being transported to the scanning room to help keep the baby calm and minimize movements during scanning. Babies were placed in a portable carriage and brought to the scanning room. Most newborns became drowsy after swaddling, which facilitated their standardized positioning, thus minimizing movement artifacts in the scanning. During scanning, infants were in supine position on the scan table. The scan began at the top of the head and moved in a rectilinear pattern down the body to the feet. The scan was stopped and repeated if there was a significant movement. BMC was measured in grams (g).

### Statistical Analysis

Statistical software SPSS for Windows, version 18.0 (IBM Corp., Chicago, IL, USA) was used to perform data analyses. Data were expressed as means ± standard deviation (SD) or ratio. The associations between WB BMC at birth and the determinants (sex, gestational age, birth weight, birth length, maternal anemia, maternal smoking exposure, and pre-pregnancy BMI) were analyzed using linear regression. The mean differences between subgroups were identified using ANOVA and *post hoc* Tukey's test. Independent *t*-test was also used to detect mean difference between two groups. Significance was set at *p* < 0.05. Because there are no data of newborn BMC available in Indonesia, we decided not to calculate sample size. We start with observational study to evaluate BMC in Indonesian newborns.

The study was approved by the Medical and Health Research Ethics Committee of the Faculty of Medicine, Public Health, and Nursing, Universitas Gadjah Mada, Yogyakarta. Written parental informed consent was obtained from both parents of the study subjects in accordance with the appropriate Ethics Committee requirements.

## Results

A total of 45 term AGA newborns consisting of 23 male and 22 female newborns were included in this study. The subjects' characteristics are shown in Table 1. The mean ± SD of the gestational age was 38.7 ± 1.0 weeks (range of 37.0–40.9 weeks), while the mean ± SD of birth weight was 3,134.1 ± 281.1 g (range 2,486 −3,745 g). Most mothers had multiparous pregnancy (60%), normal pre-gestational nutritional status (60%), and no cigarette exposure (82.2%). All mothers with cigarette exposure were passive smokers. Most mothers had no anemia (55.6%). The results of BMC scans were showed in Table 2.

**Table 1:** Baseline characteristics of the study subjects (*N* = 45).

**Characteristics**	**Mean ± SD or N (%)**
**Maternal**	
Age (years)	32.1 ± 6.1
Parity^a^	
Primipara	18 (40.0%)
Multipara	27 (60.0%)
Height (m)	1.56 ± 0.05
Pre-gestational BMI (kg/m^2^)	23.8 ± 4.1
Pre-gestational Nutritional Status (*N*)	
Underweight (BMI <18.5)	3 (6.7%)
Normal (18.5 < BMI <24.99)	27 (60.0%)
Overweight (25 < BMI <29.99)	11 (24.4%)
Obese (BMI ≥ 30)	4 (8.9%)
Anemia (*N*)	
Yes (hemoglobin <6.83 mmol/l)	20 (44.4%)
No (hemoglobin ≥ 6.83 mmol/l)	25 (55.6%)
Education (*N*)	
University	24 (53.3%)
High school and lower	21 (46.7%)
Cigarette exposure	
Yes	8 (17.8%)
No	37 (82.2%)
**Neonatal**	
Gender (*N*)	
Male	23 (51.1%)
Female	22 (48.9%)
Birth weight (g)	3134.1 ± 281.1
Birth length (cm)	49.4 ± 1.8
Head circumference (cm)	34.1 ± 1.4
Gestational age (weeks)	38.7 ± 1.0
Age of DXA measurement (days)	3.6 ± 2.7

a *Parity: the number of times that the mother has given birth; primipara: mother has given birth once; multipara: mother given birth more than once*.

*BMI, body mass index*.

**Table 2:** Mean ± SD BMC of whole body, whole body except head, trunk and legs.

**Site**	**Mean ± SD**
WB BMC (g)	33.2 ± 9.3
WB BMC except head (g)	13.7 ± 5.0
Trunk BMC (g)	6.2 ± 2.7
Legs BMC (g)	6.7 ± 7.0

*BMC, bone mineral content; WB BMC, whole body BMC*.

WB BMC was significantly higher in male infants compared with female infants (*p* = 0.028). Infants born to mothers exposed to cigarette smoke had a significantly lower WB BMC than those not exposed (*p* = 0.046) (Table 3).

**Table 3:** Mean of bone mineral content according to determinant variables.

**Characteristics**	**WB BMC (mean ± SD)**	***P*-value**
**Sex**		
Male (23)	36.2 ± 9.3	0.028^a^
Female (22)	30.2 ± 8.3	
**Maternal cigarette exposure**		
Yes (8)	27.3 ± 9.1	0.046^a^
No (37)	34.5 ± 8.9	
**Pre-gestational nutritional status**		
Underweight (BMI <18.5) (3)	27.5 ± 5.7	0.336^b^
Normal (18.5 < BMI <24.99) (27)	34.9 ± 10.0	
Overweight (25 < BMI <29.99) (11)	29.9 ± 8.7	
Obese (BMI ≥ 30) (4)	34.5 ± 2.4	

a *p-value for independent sample t-test*.

b *p-value for one-way Anova*.

*BMI, body mass index; WB BMC, whole body bone mineral content*.

Linear regression analysis showed that sex, gestational age, birth weight, birth length and maternal cigarette exposure were significantly associated with WB BMC at birth (Table 4).

**Table 4:** Determinants of WB BMC at birth by linear regression analysis.

**Independent variable**	**Simple linear regression analysis**				**Multiple linear regression analysis**			
	**β**	95% CI*	***p***	**R^**2**^**	**β**	95% CI*	***p***	**Adjusted R^**2**^**
Sex (0 = female, 1 = male)	5.93	0.56–11.31	0.031	0.10	4.52	0.61–8.44	0.025	0.55
Gestational age (weeks)	4.36	1.96–4.78	0.001	0.24	4.24	2.32–6.16	<0.001	
Birth weight (g)	0.02	0.01–0.03	<0.001	0.25	0.01	0.00–0.02	0.048	
Birth length (cm)	2.27	0.33–4.21	0.023	0.12	1.78	0.34–3.22	0.017	
Maternal hemoglobin level (g/dl)	−1.07	−2.62–0.48	0.270	0.02				
Maternal cigarette exposure (0 = no, 1 = yes)	−7.13	−14.22–(-0.04)	0.049	0.09	−6.48	−11.47–(−1.48)	0.012	
Maternal pre-gestational BMI (kg/m^2^)	−0.22	−0.92–0.47	0.523	0.01				
Maternal height (m)	18.55	−37.77–74.88	0.510	0.01				
Maternal education (0 = University,1 = Senior high school and lower)	−1.34	−7.01–4.33	0.636	0.01				

*CI, confidence interval for β; BMI, body mass index*.

Multiple linear regression analysis was also performed to assess which variables were independently associated with WB BMC. Variables with *p* < 0.25 in the simple regression analysis were included in the multiple regression model (18). The results of the multivariate linear regression analysis showed that neonatal factors, that is, birth weight, birth length, and gestational age were positively and independently associated with WB BMC (*p* = 0.048, 0.017, and <0.001, respectively). Male infants had significantly higher of WB BMC than female (*p* = 0.025). On the other hand, only maternal smoking exposure was negatively associated the newborns' WB BMC (*p* = 0.012). Those determinants contribute to almost 50% of the variance of WB BMC (adjusted *R*^2^ = 0.55).

## Discussion

The present study investigated the association between maternal and neonatal conditions and BMC assessed with DXA in term AGA Indonesian newborns at birth. The results of our study showed that birth weight, -length, gestational age, sex, and maternal smoking exposure had significant associations with BMC.

The International Society for Clinical Densitometry (ISCD) advises that spine and whole body scans be used to diagnose bone disorders in children. However, there are currently no clear guidelines for the infant. In infants, measuring the whole body except head may be preferred because of inaccuracies in the algorithm for determining the BMC of the skull. Spine vertebrae especially the lumbar is recommended because of their speed and precision of measurement, easily identified bony landmarks, and less subjected to movement artifact. However, because of its excellent precision and the fact that it measures overall bone density, the total body is still a preferred site (19). Moreover, data from an earlier study conducted by Gallo and colleagues showed that the WB BMC measurement data up to 6 months were robust and consistent with other studies (20).

The WB BMC observed in this study was much lower than the total body BMC observed in several studies conducted in developed countries. A systematic review of term infants, mostly from developed countries, found a mean WB BMC of 68.8 g and 65.9 g for male and female infants, respectively (2). This systematic review also reported that the highest WB BMC was seen in newborns from North America followed by Europe and Africa while Asian newborns had the lowest WB BMC (2).

It is well-known that vitamin D is essential for mineral-bone homeostasis. Several studies have reported that maternal serum 25-hydroxyvitamin D (25 [OH] D) concentrations are associated with offspring bone mass (21–23). Low levels of vitamin D in pregnant women may be one of the causes of low WB BMC in neonates in Indonesia. A study conducted in the same setting as our present study (Yogyakarta) detected vitamin D deficiency in 90% of cord blood samples (24). Two others studies conducted in two different places in Indonesia found that the prevalence of vitamin D deficiency in early pregnancy was very high, namely in Minangkabau: 82.8% and in Jakarta: 99.6% (9, 10). A study in Korea observed that in winter, maternal low vitamin D status resulted in a marked reduction in WB BMC of the newborn (25). However, a more recent randomized clinical trial reported that vitamin D supplementation during pregnancy did not increase offspring WB BMC compared with placebo (26). Unfortunately, maternal 25 (OH) D levels were not measured in our study.

The supply of calcium from maternal sources plays an important role in the normal development of fetal bones. Maternal hypocalcemia may stimulate intrauterine parathyroid hyperplasia causing poor mineralization of the fetal skeleton (27). Previous research conducted in two different places in Indonesia, that is, Java and Sumatra, shows that the calcium intake of pregnant women in both areas was below the recommended daily allowance (28, 29).

As observed in previous research, the findings of the present study found an increase in WB BMC that was consistent with rising birth weight and length (5, 30). This makes sense because WB BMC is dependent on the size and density of the bone, and WB BMC differences can represent bone size and density differences (31).

Our findings are consistent with the results of a previous study indicating that birth weight and birth length were strongly, positively associated with neonatal bone mass even after adjusting for sex and gestational age (32). In the last trimester of pregnancy, a dramatic increase in calcium transfer across the placenta to fetal circulation will be followed by increased fetal bone mineralization. In a healthy term human fetus, two-thirds of total body calcium is transported during the last trimester. The peak accretion is between 36 and 38 weeks of gestation (33, 34). Hence, under normal uteroplacental circumstances, the more mature the fetus, the better the bone mineralization. Data also showed that birth weight also predicts later bone mass (35).

Our results indicate that there is a significant difference in WB BMC between male and female term infants, in which male infants have a higher WB BMC than female infants. These findings are consistent with previous studies of total body composition which showed that males had relatively more lean and less fat mass than females (36) and bone is a lean body component. The difference in body composition between male and female fetuses is most likely influenced by sex steroid hormones. *In utero*, the testes produce testosterone, which is believed to increase lean body mass in fetal life. In the first week of life, male infant testosterone levels increase rapidly and nearly match the concentrations achieved in adult males, whereas the ovaries are relatively silent regarding estrogen production, and do not release significant amounts of testosterone during perinatal development (36).

The differences in WB BMC between male and female term infants at birth are, however, still conflicting. Several studies have shown that males have a significantly higher BMC at birth than female infants (6, 37–39). Meanwhile, several other studies at birth observed no difference in male and female newborns WB BMC (5, 16, 35). Data in adults showed that the incidence of osteoporosis and fractures in women is higher than in men, and this is because the bone mass of women is lower than that of men. Data also suggested that these differences might already occur early in life (40). Namgung and colleagues reported that in term infants there were differences in WB BMC between males and females at the beginning of life but not at birth (5). This phenomenon was also supported by other research which found that male infants aged 1–18 months had higher total body bone mass than female infants, even after controlling for weight, length and race (6).

Another important finding in this study is the effect of smoking exposure on bone mineralization. In this study, all respondents who reported exposure to cigarette smoke were not active smokers. This finding is in line with national data, which showed that prevalence of smoking among women in Indonesia was much lower than that of men (11). The results of our study indicated that exposure to cigarette smoke during pregnancy had a detrimental effect on neonatal WB BMC even though these pregnant women were not active smokers. A cohort study conducted in Finland showed the harmful effects of passive smoking in children on their bone health in adulthood (41).

The negative effects of smoking during pregnancy on neonatal bone mineralization had been demonstrated by several previous studies (35, 42). Evidence from an animal study on 4-week-old mice whose mothers were exposed to cigarette smoke observed that cigarette exposure during pregnancy had a negative influence on bone microarchitecture of the offspring (42).

Several possible mechanisms have been proposed to explain the relationship between maternal smoking and infant bone mass. Maternal smoking has detrimental effect on placental function, causing impairment of uteroplacental blood flow and leading to reduce fetal oxygen carrying capacity. Disruption of the placenta will also cause disruption of mineral transport from mother to fetus. Since fetal bone mineralization is determined by the transfer of placental minerals, fetal bone mineral accretion rate and the rate at which fetal bone is resorbed, impaired mineral transport will cause disruption of fetal bone mineralization (5, 43). Another possible mechanism is due to the high concentration of cadmium as a tobacco smoke contaminant which has specific effects on osteoblast function and on trophoblast calcium transport. This condition may interfere with fetal bone development (16, 35). Exposure to cigarette smoke in pregnant women is also suspected to decrease calcium absorption and lead to bone demineralization. Indirectly, this can affect fetal bone mineralization (30).

However, some results from studies investigating the relationship between maternal smoking with offspring's bone mineralization are inconclusive (44). A study published in 1991 found that in full-term infants, bone mineralization was not significantly altered by maternal cigarette smoking. However, this study showed the limitation that the bone mineralization was examined at one third of the distal of the radius (30). This site is not recommended by the ISCD, as there are few normative data for pediatric forearm studies, and the values obtained may only be useful when compared with subsequent studies (19, 45). A recent systematic review aimed at exploring the effect of maternal smoking during pregnancy on the bone mineral density (BMD) of children or neonates concluded that smoking during pregnancy has no direct effect on BMD of the offspring. Other factors such as placental weight, birth weight, and present body size of children may confound the relation between BMD and smoking during pregnancy (46).

One of the weaknesses in research on maternal smoking or exposure to cigarette smoke during pregnancy is that the data on smoking exposure were obtained from subjective measurements such as a self-reporting questionnaire (46). This is also the case in our study.

## Strengths and Limitations

To our knowledge our study is the first attempt to use DXA in an Indonesian population to measure bone mass in term newborns. The availability of instruments to assess BMC is one of the barriers to conducting research of bone mass in newborns in developing countries. Furthermore, bone mass measurement has often been an area of debate, with many researchers using various methods to report bone mass. However, DXA has been considered to be the gold standard for bone mass measurement and is used in both clinical and academic studies. DXA is also an ideal method because radiation exposure is low and scan time is fast (2).

The small sample size of the research will limit the generalizability of the findings of this study. This disadvantage can, however, be accounted for since all of the study participants were of Javanese origins, which is Indonesia's largest ethnicity. Another limitation of this study is its cross-sectional study design which makes it difficult to assess if our findings are only a temporal effect.

## Conclusions

The WB BMC in our study population was lower than other populations in developed countries. Birth weight, -length, gestational age, sex, and maternal cigarette exposure during pregnancy were independently and significantly associated with WB BMC.

## Data Availability Statement

The datasets presented in this article are not readily available because the dataset is part of neonatal body composition research and will be analyzed for other publications. Requests to access the datasets should be directed to tunjungwibowo@ugm.ac.id.

## Ethics Statement

The studies involving human participants were reviewed and approved by Medical and Health Research Ethics Committee (MHREC) Faculty of Medicine Gadjah Mada University-Dr. Sardjito General Hospital. Written informed consent to participate in this study was provided by the participants' legal guardian/next of kin.

## Author Contributions

TW performed the conception and investigation of the study, performed the statistical analysis, prepared the tables, and wrote the final version of the manuscript. NN identified the statistical methods and validated the statistical analysis results. JH reviewed and edited the final version of the manuscript and did the verification of the reproducibility results. AA helped the investigation of the study and maintained the research data. MH, MJ, and MV obtained funding for the study, advised the study design, supervised the statistical analysis, interpretation of the results, and reviewed the final version of the manuscript. All authors contributed to the article and approved the submitted version.

## Conflict of Interest

The authors declare that the research was conducted in the absence of any commercial or financial relationships that could be construed as a potential conflict of interest.

## References

[1] Harvey N Dennison E Cooper C. Osteoporosis: impact on health and economics. Nat Rev Rheumatol. (2010) 6:99–105. 10.1038/nrrheum.2009.260 20125177

[2] Ramot R Kachhawa G Kulshreshtha V Varshney S Sankar MJ Devasenathipathy K. Bone mass in newborns assessed by DXA—a systematic review and meta-analysis. Indian J Endocrinol Metab. (2019) 23:198–205. 10.4103/ijem.IJEM_681_18 31161103 PMC6540894

[3] Godfrey KM. Maternal regulation of fetal development and health in adult life. Eur J Obstet Gynecol Reprod Biol. (1998) 78:141–50. 10.1016/S0301-2115(98)00060-8 9622311

[4] Minton SD Steichen JJ Tsang RC. Bone mineral content in term and preterm appropriate-for-gestational-age infants. J Pediatr. (1979) 95:1037–42. 10.1016/S0022-3476(79)80305-4 501482

[5] Namgung R Tsang RC. Factors affecting newborn bone mineral content: *In utero* effects on newborn bone mineralization. Proc Nutr Soc. (2000) 59:55–63. 10.1017/S0029665100000070 10828174

[6] Rupich RC Specker BL Lieuw-A-Fa M Ho M. Gender and race differences in bone mass during infancy. Calcif Tissue Int. (1996) 58:395–7. 10.1007/BF02509436 8661478

[7] Horlick M Thornton J Wang J Levine LS Fedun B Pierson RN. Bone mineral in prepubertal children: Gender and ethnicity. J Bone Miner Res. (2000) 15:1393–7. 10.1359/jbmr.2000.15.7.1393 10893689

[8] Kruger MC Todd JM Schollum LM Kuhn-Sherlock B McLean DW Wylie K. Bone health comparison in seven Asian countries using calcaneal ultrasound. BMC Musculoskelet Disord. (2013) 14:1–9. 10.1186/1471-2474-14-81 23497143 PMC3602652

[9] Wibowo N Bardosono S Irwinda R Syafitri I Putri AS Prameswari N. Assessment of the nutrient intake and micronutrient status in the first trimester of pregnant women in Jakarta. Med J Indones. (2017) 26:109–15. 10.13181/mji.v26i2.1617

[10] Aji AS Erwinda E Yusrawati Y Malik SG Lipoeto NI. Vitamin D deficiency status and its related risk factors during early pregnancy: A cross-sectional study of pregnant Minangkabau women, Indonesia. BMC Pregnancy Childbirth. (2019) 19:1–10. 10.1186/s12884-019-2341-4 31117971 PMC6532131

[11] RISKESDAS. Indonesia Basic Health Survey. Jakarta (2018). Available online at: http://www.yankes.kemkes.go.id/assets/downloads/PMK No. 57 Tahun 2013 tentang PTRM.pdf (accessed November 01, 2020).

[12] Institute of Medicine and National Research Council. In: Rasmussen KM Yaktine AL editors. Weight Gain During Pregnancy: Reexamining the Guidelines. Washington, DC: National Academies Press (2009). 20669500

[13] Hamaguchi Y Kondo T Nakai R Ochi Y Okazaki T Uchihashi K. Introduction of products over view and features of the automated hematology analyzer XN-L series. Sysmex J Int. (2015) 25:1–12.

[14] WHO UNICEF UNU. Iron Deficiency Anaemia: Assessment, Prevention, and Control, A Guide for Programme Manager.. Geneva: WHO, UNICEF, UNU (2001).

[15] Chou JH Roumiantsev S Singh R. PediTools electronic growth chart calculators: applications in clinical care, research, and quality improvement. J Med Internet Res. (2020) 22:e16204. 10.2196/16204 32012066 PMC7058170

[16] Beltrand J Alison M Nicolescu R Verkauskiene R Deghmoun S Sibony O. Bone mineral content at birth is determined both by birth weight and fetal growth pattern. Pediatr Res. (2008) 64:86–90. 10.1203/PDR.0b013e318174e6d8 18391851

[17] Cooper C Harvey N Javaid K Hanson M Dennison E. Growth and bone development. Nestle Nutr Work Ser Pediatr Progr. (2008) 61:53–66. 10.1159/000113170 18196944

[18] Mickey RM Greenland S. The impact of confounder selection criteria on effect estimation. Am J Epidemiol. (1989) 130:1066. 10.1093/oxfordjournals.aje.a115409 2910056

[19] Gordon CM Bachrach LK Carpenter TO Crabtree N El-Hajj Fuleihan G Kutilek S. Dual energy X-ray absorptiometry interpretation and reporting in children and adolescents: The 2007 ISCD pediatric official positions. J Clin Densitom. (2008) 11:43–58. 10.1016/j.jocd.2007.12.005 18442752

[20] Gallo S Vanstone CA Weiler HA. Normative data for bone mass in healthy term infants from birth to 1 year of age. J Osteoporos. (2012) 2012:672403. 10.1155/2012/672403 23091773 PMC3468026

[21] Harvey NC Holroyd C Ntani G Javaid K Cooper P Moon R. Vitamin D supplementation in pregnancy: a systematic review. Health Technol Assess. (2014) 18:1–189. 10.3310/hta18450 25025896 PMC4124722

[22] Ioannou C Javaid MK Mahon P Yaqub MK Harvey NC Godfrey KM. The effect of maternal vitamin D concentration on fetal bone. J Clin Endocrinol Metab. (2012) 97:E2070–7. 10.1210/jc.2012-2538 22990090 PMC3485609

[23] Viljakainen HT Saarnio E Hytinantti T Miettinen M Surcel H Mäkitie O. Maternal Vitamin D Status Determines Bone Variables in the Newborn. J Clin Endocrinol Metab. (2010) 95:1749–57. 10.1210/jc.2009-1391 20139235

[24] Oktaria V Graham SM Triasih R Soenarto Y Bines JE Ponsonby A. The prevalence and determinants of vitamin D deficiency in Indonesian infants at birth and six months of age. PLoS ONE. (2020) 15:e0239603. 10.1371/journal.pone.0239603 33017838 PMC7535980

[25] Namgung R Tsang RC Lee C Han DG Ho ML Sierra RI. Low total body bone mineral content and high bone resorption in Korean winter-born versus summer-born newborn infants. J Pediatr. (1998) 132:421–5. 10.1016/S0022-3476(98)70013-7 9544894

[26] Cooper C Harvey NC Bishop NJ Kennedy S Papageorghiou AT Schoenmakers I. Maternal gestational vitamin D supplementation and offspring bone health (MAVIDOS): A multicentre, double-blind, randomised placebo-controlled trial. Lancet Diabetes Endocrinol. (2016) 4:393–402. 10.1016/S2213-8587(16)00044-9 26944421 PMC4843969

[27] Thomas AK McVie R Levine SN. Disorders of maternal calcium metabolism implicated by abnormal calcium metabolism in the neonate. Am J Perinatol. (1999) 16:515–20. 10.1055/s-1999-7280 10874987

[28] Setiarsih D Wirjatmadi B Adriani M. Bone density status and vitamin D and calcium concentrations in pregnant and non-pregnant women. Makara J Heal Res. (2016) 20:63–8. 10.7454/msk.v20i3.3540

[29] Aji AS Yerizel E Desmawati D Lipoeto NI. Low maternal vitamin d and calcium food intake during pregnancy associated with place of residence: a cross-sectional study in west sumatran women, Indonesia. Open Access Maced J Med Sci. (2019) 7:2879–85. 10.3889/oamjms.2019.659 31844453 PMC6901836

[30] Venkataraman PS Duke JC. Bone mineral content of healthy, full-term neonates: effect of race, gender, and maternal cigarette smoking. Am J Dis Child. (1991) 145:1310–2. 10.1001/archpedi.1991.02160110102030 1951227

[31] Mølgaard C Thomsen BL Michaelsen KF. Whole body bone mineral accretion in healthy children and adolescents. Arch Dis Child. (1999) 81:10–15. 10.1136/adc.81.1.10 10373125 PMC1717991

[32] Cooper C Harvey N Cole Z Hanson M Dennison E. Developmental origins of osteoporosis: the role of maternal nutrition. Adv Exp Med Biol. (2009) 646:31–9. 10.1007/978-1-4020-9173-5_3 19536660

[33] Namgung R Tsang RC. Bone in the pregnant mother and newborn at birth. Clin Chim Acta. (2003) 333:1–11. 10.1016/S0009-8981(02)00025-6 12809730

[34] Abrams SA. Normal acquisition and loss of bone mass. Horm Res. (2003) 60:71–6. 10.1159/000074505 14671401

[35] Godfrey K Walker-Bone K Robinson S Taylor P Shore S Wheeler T. Neonatal bone mass: Influence of parental birthweight, maternal smoking, body composition, and activity during pregnancy. J Bone Miner Res. (2001) 16:1694–703. 10.1359/jbmr.2001.16.9.1694 11547840

[36] Simon L Borrego P Darmaun D Legrand A Rozé JC Chauty-Frondas A. Effect of sex and gestational age on neonatal body composition. Br J Nutr. (2013) 109:1105–8. 10.1017/S0007114512002991 22784704

[37] Holroyd CR Harvey NC Crozier SR Winder NR Mahon PA Ntani G. Placental size at 19 weeks predicts offspring bone mass at birth: Findings from the Southampton Women's Survey. Placenta. (2012) 33:623–9. 10.1016/j.placenta.2012.04.007 22640438 PMC3800076

[38] Harvey NC Javaid MK Arden NK Poole JR Crozier SR Robinson SM. Maternal predictors of neonatal bone size and geometry: The Southampton Women's Survey. J Dev Orig Health Dis. (2010) 1:35–41. 10.1017/S2040174409990055 23750315 PMC3672833

[39] Javaid MK Godfrey KM Taylor P Robinson SM Crozier SR Dennison EM. Umbilical cord leptin predicts neonatal bone mass. Calcif Tissue Int. (2005) 76:341–7. 10.1007/s00223-004-1128-3 15864467

[40] Gilsanz V Kovanlikaya A Costin G Roe TF Sayre J Kaufman F. Differential Effect of Gender on the Sizes of the Bones in the Axial and Appendicular Skeletons^*^. J Clin Endocrinol Metab. (1997) 82:1603–7. 10.1210/jc.82.5.1603 9141557

[41] Juonala M Pitkanen N Tolonen S Laaksonen M Sievanen H Jokinen E. Childhood exposure to passive smoking and bone health in adulthood: The cardiovascular risk in young finns study. J Clin Endocrinol Metab. (2019) 104:2403–11. 10.1210/jc.2018-02501 30715377

[42] Cho GJ Sim JY Kim SE Hong H-R Ahn K-H Hong S-C. Maternal Smoke during Pregnancy Programs for Bone Disturbance in Offspring. Perinatology. (2018) 29:114. 10.14734/PN.2018.29.3.114

[43] Kovacs CS. Bone development and mineral homeostasis in the fetus and neonate: roles of the calciotropic and phosphotropic hormones. Physiol Rev. (2014) 94:1143–218. 10.1152/physrev.00014.2014 25287862

[44] Brand JS Hiyoshi A Cao Y Lawlor DA Cnattingius S Montgomery S. Maternal smoking during pregnancy and fractures in offspring: National register based sibling comparison study. BMJ. (2020) 368:1–10. 10.1136/bmj.l7057 31996343 PMC7190030

[45] Binkovitz LA Henwood MJ. Pediatric DXA: Technique and interpretation. Pediatr Radiol. (2007) 37:21–31. 10.1007/s00247-006-0153-y 16715219 PMC1764599

[46] Baradaran Mahdavi S Daniali SS Farajzadegan Z Bahreynian M Riahi R Kelishadi R. Association between maternal smoking and child bone mineral density: a systematic review and meta-analysis. Environ Sci Pollut Res. (2020) 27:23538–49. 10.1007/s11356-020-08740-1 32314283

